# Combined Aerobic Training and Mediterranean Diet Is Not Associated with a Lower Prevalence of Sarcopenia in Italian Older Adults

**DOI:** 10.3390/nu15132963

**Published:** 2023-06-29

**Authors:** Hélio José Coelho-Júnior, Riccardo Calvani, Anna Picca, Stefano Cacciatore, Matteo Tosato, Francesco Landi, Emanuele Marzetti

**Affiliations:** 1 Department of Geriatrics, Orthopedics, and Rheumatology, Università Cattolica del Sacro Cuore, 00168 Rome, Italy; riccardo.calvani@unicatt.it (R.C.); francesco.landi@unicatt.it (F.L.); 2 Fondazione Policlinico Universitario “A. Gemelli”, IRCCS, 00168 Rome, Italy; picca@lum.it (A.P.); matteo.tosato@policlinicogemelli.it (M.T.); 3 Department of Medicine and Surgery, LUM University, 70100 Casamassima, Italy

**Keywords:** muscle strength, frailty, exercise training, nutrition, diet patterns, elderly

## Abstract

Previous studies found a lower prevalence of sarcopenia in older adults engaged in regular aerobic training (AT) or with greater adherence to a Mediterranean (MED) diet. However, the effect of their combination on sarcopenia indices is unknown. The present study tested the association between AT plus a MED diet and the presence of sarcopenia and its defining elements in a sample of Italian older adults enrolled in the Longevity Check-up 7+ (Lookup 7+) project. Analyses were conducted in participants 65+ years, with a body mass index of at least 18.5 kg/m^2^, engaged in regular AT, and without missing information for the variables of interest. MED diet adherence was evaluated via a modified version of the MEDI-LITE score and categorized as low, moderate, or high. The presence of sarcopenia was established by handgrip strength and appendicular skeletal muscle mass (ASM) values below sex-specific cut-points recommended by the European Working Group on Sarcopenia in Older People 2. Data from 491 older adults were analyzed for the present study. The mean age was 72.7 ± 5.7 years, and 185 (37.7%) were women. MED diet adherence was low in 59 (12.0%) participants, moderate in 283 (57.6%), and high in 149 (30.3%). Sarcopenia was identified in 26 participants (5.3%), with no differences across MED diet adherence groups. The results of binary logistic regression showed no significant associations between AT plus adherence to a MED diet and dynapenia, low ASM, or sarcopenia. The findings of the present study indicate that the combination of AT with a MED diet is not associated with a lower probability of sarcopenia or its defining elements in Italian older adults enrolled in Lookup 7+. Further research is warranted to establish whether exercise frequency, volume, intensity, and length of engagement in AT impact the association between MED diet and sarcopenia.

## 1. Introduction

Sarcopenia is a neuromuscular disease that involves a supraphysiologic loss of muscle mass and strength [1,2], while a reduced physical function is an indicator of severity [1,2]. The prevalence of sarcopenia increases with age and exceeds 20% in those 65+ years [3]. Sarcopenia is associated with numerous negative health outcomes, including falls, hospitalization, admission to residential care, and death [4]. The progression of sarcopenia is also frequently associated with the occurrence of other health conditions (e.g., malnutrition, frailty, high blood pressure) [4,5,6,7].

Exercise training, a combination of organized motor tasks that aims to develop or maintain one or more attributes of physical fitness [8], is indicated as a first-line intervention to counteract sarcopenia development and progression [9,10,11]. Resistance training, a form of exercise in which muscles contract to support or move a load [8], has been shown to enhance sarcopenia-related parameters in older persons [12,13]. A recent network meta-analysis found that older adults with sarcopenia who engaged in resistance training programs had greater gains in muscle strength and function compared with other exercise modalities [14].

Aerobic training (AT), a form of exercise where muscle contractions are maintained for extended periods of time [8], has also been tested against sarcopenia. Although promising findings have recently been reported [15], pooled analyses indicated limited effects of this type of intervention on sarcopenia-related parameters [16]. The subject deserves further exploration, given that AT is an effective strategy for the prevention and management of cardiovascular and metabolic diseases [17,18,19,20]. Furthermore, land- and water-based AT programs can be integrated into ludic activities (e.g., water volleyball) [21], which might foster adherence and participant well-being.

The Mediterranean (MED) diet refers to a dietary pattern passed down through many generations resulting from centuries of cultural interactions among inhabitants of the MED region [22,23]. The traditional MED diet pyramid involves a high intake of plant foods (e.g., fruits, vegetables, legumes, grains, nuts), moderate amounts of eggs, seafood, and dairy products, and occasional consumption of meat [22,23]. The cardioprotective properties of the MED diet have long been recognized [24,25]. More recently, a higher MED diet adherence has been associated with better physical performance in older adults [26]. In addition, the MED diet has been negatively associated with the presence of sarcopenia [27]. These premises suggest that older adults who practice AT and have moderate or high adherence to a MED diet might show a lower prevalence of sarcopenia relative to physically active peers with low MED diet adherence.

The Longevity Check-Up 7+ (Lookup 7+) project is an ongoing study that started in June 2015. The objective of Lookup 7+ is to promote the adoption of healthy lifestyles by the general Italian population to lower the prevalence of risk factors for chronic diseases. The Lookup 7+ database offered the possibility to explore, in a “real world” context, several health parameters relevant to clinicians and researchers, including the prevalence of cardiometabolic risk factors [28] and their association with physical function [29] and anthropometric measures [30,31]. The adherence to physical activity recommendations and the practice of different types of exercise training have also been examined [32].

Two prior studies by our group explored the relationship between physical exercise or adherence to a MED diet and sarcopenia in older adults enrolled in Lookup 7+ [15,27]. In one study, we investigated the association between adherence to specific exercise modalities and the presence of sarcopenia or severe sarcopenia [15]. Sarcopenia was operationalized based on the cooccurrence of low appendicular skeletal muscle mass (ASM) and low muscle strength of upper or lower extremities estimated by isometric handgrip (IHG) strength and five-time sit-to-stand tests, respectively. Information on self-reported mobility limitations was used to define the severity of sarcopenia. Analyses were conducted in 3289 participants (mean age: 72.7 years, standard deviation (SD) ± 5.7 years; 55.2% women) and showed that engagement in AT was negatively associated with sarcopenia in both men and women, with sex-specific patterns [15]. In another study, we explored whether the level of adherence to a MED diet was associated with probable sarcopenia, operationalized as an IHG strength below sex-specific cut-points recommended by the European Working Group on Sarcopenia in Older People 2 (EWGSOP2) [2,27]. We found that a high MED diet adherence was indeed associated with lower odds of probable sarcopenia in 2963 older adults (mean age 72.8 years, SD ± 5.7 years; 54.4% women).

Altogether, these observations suggest that the negative association between AT and sarcopenia might be amplified in older adults with a high MED diet adherence. This possibility was explored in the present study by analyzing associations between the practice of AT plus different levels of adherence to a MED diet and low IHG strength, low ASM, and sarcopenia in older adults enrolled in Lookup 7+.

## 2. Materials and Methods

Data analyzed for the present study were obtained from the Lookup 7+ database. Participants were recruited in unconventional contexts, including exhibitions, shopping centers, and social gatherings, or during prevention campaigns organized by our university. This recruitment strategy was adopted to include relatively unselected participants outside of the usual contexts for medical care or scientific research. The manuscript was written in accordance with the Strengthening the Reporting of Observational Studies in Epidemiology (STROBE) guidelines for observational studies [33].

### 2.1. Participants

From 1 June 2015 to 31 March 2023, 13,515 adults living in the community were enrolled in the Lookup 7+ project. Exclusion criteria were self-reported pregnancy, difficulty or incapacity to perform the physical function tests required by the protocol, and refusal or inability to provide written informed consent. For the present study, analyses were performed using information from participants (*n* = 491) who were 65 or older, had a body mass index (BMI) of at least 18.5 kg/m^2^, practiced regular AT, and had no missing data for the variables of interest.

### 2.2. Data Collection

Data on dietary and exercise habits and other lifestyle behaviors were collected using a structured interview. Smoking status was divided into current (defined as having smoked 100 or more cigarettes in their lifetime and still doing so) and non-current smoker categories. A standard stadiometer and an analog scale were used to measure standing height and body weight, respectively. The BMI was determined as the ratio between body weight (kg) and the square of height (m^2^). Regular physical activity was defined as engaging in leisure activities at least twice a week, for a minimum of 30 minutes each time, in the prior year [32]. Regular engagement in AT was operationalized as the practice of aerobic activities (e.g., running, swimming) at least twice weekly for a minimum of 30 minutes per session during the previous year [32,34]. The interview did not include questions about exercise frequency, volume, intensity, or length of engagement in AT.

### 2.3. Mediterranean Diet Adherence

Nutrition data were gathered through the administration of a food frequency questionnaire. A modified version of the MEDI-LITE score was used to evaluate adherence to a MED diet, as previously described [27]. The instrument is based on the frequency of consumption of nine food groups: (1) fruit, (2) vegetables, (3) cereals, (4) legumes, (5) fish and seafood, (6) meat and derivatives, (7) dairy products, (8) alcohol, (9) olive oil. Food items typically included in a MED diet (i.e., fruit, vegetables, grains, legumes, and fish) received a score from 2 (highest intake) to 0 (lowest consumption). Meat, meat derivatives, and dairy products, which are not included in a typical MED diet, were given 0 points for the highest intake, 1 point for intermediate consumption, and 2 points for the lowest consumption. For olive oil, a score of 2 points was assigned to daily consumption, 1 point to frequent use, and 0 points to infrequent use. Information on alcohol intake was not collected, and the item was not scored. Thus, the maximum MEDI-LITE score was 16 rather than 18. A high MED diet adherence was operationalized as a MEDI-LITE score of 12 or greater. Moderate MED diet adherence was operationalized as a score between 9 and 11, while low adherence was defined as a score of 8 or lower.

### 2.4. Operationalization of Sarcopenia

Sarcopenia was operationalized as the coexistence of dynapenia and low ASM [1,2]. IHG was used as a measure of muscle strength. Participants took the IHG test while seated in a chair with their shoulders in a neutral position. The thumb was up, the hand was neutral, and the elbow was flexed at 90 degrees close to the body on the arm that was being assessed. The maximum contraction was measured using a portable hydraulic dynamometer (North Coast Hydraulic Hand Dynamometer; North Coast Medical, Inc., Morgan Hill, CA, USA). One familiarization trial was performed before the test. For the analysis, the greater reading (in kg) from two IHG measurements was considered. ASM was estimated from the calf circumference of the dominant leg. The measurement was made at the largest circumference between the ankle and knee joints with participants sitting on a chair. ASM was calculated according to the formula [35]:

ASM = −10.427 + (calf circumference (cm) × 0.768) − (age × 0.029) + (sex (male = 1, female = 0)) × 7.523

Sex-specific cut-points by EWGSOP2 were used to categorize muscle strength and ASM as low [1,2].

### 2.5. Statistical Analysis

The Shapiro–Wilk test was employed to confirm that the data distribution was normal. Categorical variables are given as exact numbers and percentages, while continuous variables are reported as mean ± SD. One-way analysis of variance (ANOVA) was used to assess differences among MED diet adherence groups. Differences in categorical variables were examined by χ^2^ statistics. Binary logistic regression was conducted to test the association between AT plus moderate or high MED diet adherence and sarcopenia status and related parameters. The final model was adjusted for age, sex, BMI, walking activity, and smoking status. All tests were two-sided, with significance set at 5% (*p* < 0.05). Confidence intervals (CIs) that contained the number of 1 were not statistically significant. All analyses were performed using the SPSS software (version 23.0, SPSS Inc., Chicago, IL, USA).

## 3. Results

The main characteristics of study participants according to categories of MED diet adherence are listed in Table 1. The mean age of the 491 participants practicing AT was 72.7 ± 5.7 years, and 185 (37.7%) were women. Mean BMI values indicated that participants were in the high-normal range. The average IHG strength values were higher than the EWGSOP2 cut points for sarcopenia, while ASM was borderline. The distribution of participants with sarcopenia was not significantly different across MED diet adherence groups.

Table 2 shows the results of the binary logistic regression for the relationship between AT plus MED adherence and sarcopenia indices. No significant associations were observed between MED adherence and dynapenia, low ASM, or sarcopenia.

## 4. Discussion

In the present study, we used the Lookup 7+ database to explore whether the combination between AT and moderate/high adherence to a MED diet would be associated with lower odds of sarcopenia than AT with a low MED diet adherence. Our findings revealed no significant relationships.

Studies have investigated the effects of combining AT and various dietary patterns on sarcopenia-related parameters with conflicting results. Markofski et al. [36] observed greater improvements in leg isokinetic extension and muscle quality in apparently healthy, independent-living older adults after a 24-week moderate-intensity AT program combined with essential amino acid supplementation compared with exercise or supplementation alone. No differences between groups were observed for lean mass. In contrast, Li et al. [37] reported that an eight-week low-to-moderate AT program combined with isolated soy protein supplementation increased lean mass, but not physical performance in young women. Fairbairn et al. [38] did not observe significant improvements in mobility in older women who adhered to a 24-week AT program plus docosahexaenoic acid supplementation. The results of a recent meta-analysis indicated that protein supplementation did not convey further gains in muscle strength or physical performance in endurance-trained older adults, although greater increases in muscle mass were observed [39].

The effects of a combined AT and MED diet on musculoskeletal parameters have been sparsely investigated, and, to the best of our knowledge, this is the first study that examined the association with sarcopenia. In one of the few investigations in the field, Pineda-Juárez et al. [40] reported that AT plus a MED diet had no effects on IHG strength in women with rheumatoid arthritis.

Our findings are somewhat unexpected, owing to the numerous studies that reported significant associations between adherence to a MED diet and measures of physical performance and muscle strength [26]. A possible explanation for our results lies in the fact that high adherence to a MED diet involves a moderate intake of high-protein food sources, such as fish (two to six servings a week), poultry, dairy products, and eggs (two to four servings a week), and a spare ingestion of meat (≤2 servings a week) [22,41].

Protein intake stimulates muscle protein synthesis by providing amino acids to support muscle growth [42,43,44]. Essential amino acids that are not produced by the human body and need to be obtained through dietary protein are necessary to activate muscle anabolism [44,45]. Most of these anabolic effects are mediated by the action of branched-chain amino acids (i.e., isoleucine, leucine, and valine), especially leucine [44,46]. Indeed, several studies reported that leucine supplementation may improve muscle mass, strength, and function [47].

Meat, fish, eggs, and dairy products are known as “myoprotective whole foods” [48] due to their protein content and potential effects on sarcopenia-related parameters [49,50,51,52]. The ingestion of approximately 110 g of beef increased muscle protein synthesis by 51% relative to the basal state [53]. Furthermore, a small observational study reported that beef meat consumption was positively associated with muscle mass in older adults [54]. A pooled analysis found significant improvements in lower-limb muscle strength following beef protein supplementation [55]. Further to this point, Marcos-Pardo et al. [56] suggested that a limited consumption of red and processed meat could explain the lack of associations between high adherence to a MED diet, according to the Prevención con Dieta Mediterránea (PREDIMED) score, and sarcopenia-related parameters. The authors also noted that eating more than one serving of red meat, hamburger, sausage, or cold cuts each day was associated with preservation of muscle strength and mass in European middle-aged and older adults [56].

Interesting results have also been reported by studies that examined dairy products. Whey and casein are two milk proteins that differ in amino acid composition as well as digestion and absorption kinetics [57,58,59,60], with anabolic effects on muscle tissue. Whey protein has a high leucine content [57,58,59,60] and stimulates muscle protein synthesis to a greater extent than casein. Indeed, supplementation with whey protein increases lean body mass, mainly if combined with exercise training [61].

Regarding fish, rats fed fish-based protein had significant improvements in muscle diameter in both fast and slow fiber types [62,63]. Data from the Seniors-ENRICA study indicates that older adults with a greater consumption of fish or dairy products were at a lower risk of functional impairment than those with lower intakes [64]. Moreover, a high consumption of eggs was associated with a reduced prevalence of sarcopenia in older Korean adults [52]. Notably, a low consumption of the foods mentioned above might also contribute to the development of sarcopenia through an insufficient intake of other nutrients (e.g., vitamin D and magnesium) [65].

An opposing view is that the MED diet allows protein requirements to be met through the consumption of plant-based foods. The MED diet focuses on plant food sources instead of animal-based foods [22], reflected by the fact that vegetable foods are located at the basis of the MED diet pyramid [22]. An increasing number of studies have investigated the association between plant-based diets and neuromuscular aspects. Coelho-Junior et al. [66] reported that, in well-functioning older adults, a high intake of plant-based protein was associated with faster walking speed than animal-derived protein. Yeung and Woo [67] added to these findings by reporting that a high vegetable protein intake was associated with lower declines in walking speed in older Chinese adults. Montiel-Rojas et al. [68] observed that favoring vegetable protein over animal-derived protein reduced the risk of sarcopenia in older European adults.

Such findings led experts in the field to propose that a high consumption of plant-based protein might properly stimulate muscle protein synthesis [69]. However, protein of plant origin is expected to provide a limited amount of essential amino acids, including leucin, and is characterized by a lower digestibility compared with protein from animal sources [70]. An experimental study reported that the ingestion of soy protein promoted lower muscle protein synthesis than whey protein in older men under resting conditions and after exercise, regardless of protein dose [71]. Furthermore, findings of the LifeAge Study indicated that the consumption of three or more portions of legumes per week was not associated with a better performance on IHG, sit-to-stand, gait speed, Timed “Up and Go!”, or Short Physical Performance Battery (SPPB) tests, greater muscle mass, or lower prevalence of sarcopenia in European middle-aged and older adults [56].

Altogether, the available evidence suggests that MED diet protein content might not be sufficient to improve sarcopenia parameters in older adults. Although some results in favor of vegetable protein have been observed in older adults [66,67,68], exercise-trained individuals may require larger amounts of protein than their physically inactive peers [72]. According to Camera [73], a high protein intake is not only required to properly stimulate muscle protein synthesis in aerobically trained individuals but is imperative to promote adequate recovery and performance, avoiding overtraining syndrome.

Another important aspect that might have influenced our results is lifestyle. The MED diet is a concept used to reflect the typical dietary habits observed in inhabitants of the MED basin, including Greece and South Italy [73]. This dietary pattern was mainly followed by people from rural societies and was associated with other potentially healthy behaviors [22,74]. Some authors have suggested that diet alone may not be sufficient for managing the complexity of chronic conditions, such as sarcopenia, and that the benefits of a MED diet might be conveyed by a combination of multiple healthy lifestyle factors [75,76]. Information on other lifestyle habits (e.g., social engagement, hydration, sleep time and quality, food preparation methods) were not collected in Lookup 7+ and should be analyzed in future studies.

The investigation of dietary patterns other than the MED diet is necessary to identify the optimal nutritional approach that amplifies the neuromuscular benefits of AT in older adults. However, the selection of possible candidates is not an easy task to accomplish. Results of a recent systematic review and meta-analysis indicated that healthy dietary patterns, created from established indexes or using a factor or cluster analysis, were not associated with better performance on IHG, sit-to-stand, balance, or SPPB tests, or with the prevalence of sarcopenia [77]. Although a higher adherence to healthy dietary patterns was linked to a lower reduction in gait speed, limitations in the studies included in the meta-analysis prevented the authors from conducting deeper analyses to identify the dietary pattern that conveyed the greatest benefits.

Healthy Eating Indexes (HEI), a set of widely accepted metrics that estimate diet quality according to the amount of culturally neutral foods (e.g., fruits and vegetables, whole and refined grains, added sugar) ingested per 1000 kcal [78], have been examined in relation to sarcopenia-related parameters. Parsons et al. [79] analyzed the database of the British Regional Heart Study (BRHS) to investigate the association between adherence to an HEI, composed of 86 food and drink items, and the development of mobility limitations (i.e., difficulty in walking 400 yards) in older men. Results indicated that men with greater adherence to the HEI at baseline were less likely to develop mobility limitations over 15 years of follow-up. In contrast, participants with a high-fat/low-fiber dietary pattern at baseline showed a greater risk of incident mobility limitations at follow-up [79]. On the other hand, Chan et al. [80] did not find significant associations between the adherence to an HEI and the incidence of sarcopenia, defined according to the Asian Working Group for Sarcopenia (AWGS) criteria [2], during four years of follow-up in older Chinese adults living in the community. A posteriori analysis indicated that men who followed a “vegetables-fruits” or a “snacks-drinks-milk products” dietary pattern had a lower risk of incident sarcopenia than those with high adherence to other nutritional regimens [80].

Other studies have focused on region-specific dietary patterns. The Nordic Diet Score (NDS) is a type of HEI that involves foods commonly consumed in Nordic countries (i.e., Denmark, Norway, Sweden, Finland, and Iceland), such as berries, root and cruciferous vegetables, rye bread, and a high fish intake [78]. Perala et al. [81] examined the association between adherence to an NDS and the 10-year risk of dynapenia, assessed according to IHG and isometric leg strength tests, in 1094 Finnish older adults. The authors observed that each unit increase in the NDS was related to greater leg and IHG strength in women but not men [81].

Our study has limitations that deserve acknowledgment. First, participants were community-dwelling older Caucasian adults who attended specific events, and extrapolations to other cohorts should be considered with caution. Second, ASM was estimated using an anthropometric measure (i.e., calf circumference). Third, the MED diet was operationalized using an adapted version of the MEDI-LITE score, and the possibility that results could be different using other validated questionaries (e.g., MedDiet score, PREDIMED score) cannot be ruled out. Fourth, information on chronic diseases (e.g., osteoarthritis) or medications (e.g., corticosteroids) that could impact musculoskeletal health was not collected. Fifth, exercise training variables (e.g., frequency, volume, intensity, length of engagement in AT) were not collected or controlled for. This limitation, albeit not negligible, is intrinsic to the design of the project and the unconventional settings where the survey is conducted. The collection of detailed information on lifestyle habits and medical history would substantially increase the duration of the assessments, which may deter many candidates from participating. Sixth, the use of IHG as an assessment tool for dynapenia has received some criticism [82], owing to its epidemiological and biomechanical aspects. Other tests, such as the sit-to-stand, seem to be a better proxy of muscle strength [82]. Hence, our results need to be confirmed in investigations using other measures of sarcopenia parameters. Seventh, the cross-sectional design of the study precludes the possibility of drawing cause-and-effect implications.

## 5. Conclusions

The findings of the present study indicate that the combination of AT and moderate or high adherence to a MED diet is not associated with a lower probability of sarcopenia in older Italian adults. Future investigations are warranted to explore whether other dietary regimens (e.g., high-protein diets) in combination with AT training offer therapeutic gain against sarcopenia. Further research is also necessary to establish whether exercise frequency, volume, intensity, and length of engagement in AT impact the association between MED diet and sarcopenia.

## Figures and Tables

**Table 1:** Main characteristics of study participants according to categories of adherence to a Mediterranean diet (*n* = 491).

	AT Plus Low MED Diet Adherence (*n* = 59)	AT Plus Moderate MED Diet Adherence (*n* = 283)	AT Plus High MED Diet Adherence (*n* = 149)	*p* Value
Age, years	71.3 ± 4.8	71.3 ± 5.3	72.2 ± 5.2	0.842
Sex, female	9, 1.8	49, 10.0	17, 3.5	0.199
BMI, kg/m^2^	26.1 ± 3.5	25.4 ± 3.4	24.9 ± 3.5	0.585
IHG strength, kg	30.2 ± 8.5	31.4 ± 10.9	30.9 ± 10.3	1.000
ASM, kg/m^2^	19.7 ± 5.3	19.5 ± 4.8	18.5 ± 4.8	0.344
Smoking, yes	21, 4.3	99, 20.2	65, 13.2	0.268
Walking, yes	56, 11.5	275, 56.4	144, 29.5	0.917
Dynapenia, yes	6, 1.2	30, 6.2	10, 2.1	0.422
Low ASM loss, yes	22, 4.5	90, 18.5	60, 12.3	0.169
Sarcopenia, yes	3, 0.9	15, 4.7	8, 2.5	0.970

Continuous data are shown as mean ± standard deviation, while binary data are reported as number, %. Abbreviations: ASM, appendicular skeletal muscle; BMI, body mass index; IHG, isometric handgrip.

**Table 2:** Association between adherence to a Mediterranean diet plus aerobic training and sarcopenia indices.

	Unadjusted OR * (95% CI)	Adjusted OR (95% CI)		Unadjusted OR * (95% CI)	Adjusted OR (95% CI)		Unadjusted OR * (95% CI)	Adjusted OR (95% CI)
Dynapenia								
AT plus Low MED diet adherence	1.00 (Reference)	1.00 (Reference)	AT plus Low MED diet adherence	1.00 (Reference)	1.00 (Reference)	AT plus Moderate MED diet adherence	1.000 (Reference)	1.00 (Reference)
AT plus Moderate MED diet adherence	0.96 (0.38, 2.44)	1.01 (0.38, 2.66)	AT plus High MED diet adherence	1.58 (0.54, 4.56)	2.43 (0.75, 7.86)	AT plus High MED diet adherence	1.63 (0.77, 3.43)	1.99 (0.77, 3.43)
Low appendicular skeletal muscle mass								
AT plus Low MED diet adherence	1.00 (Reference)	1.00 (Reference)	AT plus Low MED diet adherence	1.00 (Reference)	1.00 (Reference)	AT plus Moderate MED diet adherence	1.00 (Reference)	1.00 (Reference)
AT plus Moderate MED diet adherence	1.26 (0.70, 2.26)	1.64 (0.69, 3.89)	AT plus High MED diet adherence	0.85 (0.45, 1.58)	1.52 (0.64, 3.62)	AT plus High MED diet adherence	0.67 (0.44, 1.02)	0.93 (0.51, 1.70)
Sarcopenia								
AT plus Low MED diet adherence	1.00 (Reference)	1.00 (Reference)	AT plus Low MED diet adherence	1.00 (Reference)	1.00 (Reference)	AT plus Moderate MED diet adherence	1.00 (Reference)	1.00 (Reference)
AT plus Moderate MED diet adherence	0.93 (0.25–3.42)	0.97 (0.19, 4.77)	AT plus High MED diet adherence	1.04 (0.26, 4.19)	0.29 (0.03, 2.78)	AT plus High MED diet adherence	1.11 (0.45, 2.73)	0.50 (0.14, 1.72)

* The model was adjusted model was adjusted for age, sex, body mass index, walking activity, and smoking status. Abbreviations: AT, aerobic training; CI, confidence interval, MED, Mediterranean, OR, odds ratio.

## Data Availability

Data are available upon reasonable request from the corresponding authors.
